# THE EFFECTS OF LISTENING WHILE DRIVING IN OLDER AND YOUNGER ADULTS

**DOI:** 10.1093/geroni/igae098.2659

**Published:** 2024-12-31

**Authors:** Katherine Bak, Frank Russo, M Kathleen Pichora-Fuller, Jennifer Campos

**Affiliations:** University of Toronto, Toronto, Ontario, Canada; Toronto Metropolitan University, Toronto, Ontario, Canada; University of Toronto, Toronto, Ontario, Canada; The KITE Research Institute, University Health Network, Toronto, Ontario, Canada

## Abstract

Driving collisions are a top cause of accidental death globally and adults aged 65+ are overrepresented. Driving is a highly complex task, requiring sensory processing, motor control, and divided attention. Driving is especially challenging when simultaneously listening to a passenger. Age-related declines in sensory (e.g., hearing) and cognitive abilities (e.g., working memory) may result in increased effort while listening. High listening effort may reduce spare cognitive capacity, thereby limiting the cognitive resources available to support driving. However, there are very few realistic and controlled experiments examining how the demands of listening while driving are managed in older adults. Therefore, the main objective of this study was to examine performance during a driving-while-listening task in older (aged 61-80 years, 15 female) and younger (aged 20-36 years, 12 female) adults with normal hearing/vision, and cognition, using a high-fidelity driving simulator. Participants completed a driving task at two different driving loads (e.g., straight in rural (low) vs. left turn in city (high)), a listening task (Connected Speech Test [CST]) at two different listening loads (+4 signal-to-noise ratio [SNR], 0 SNR), and both tasks together, to examine dual-task costs. Results demonstrated that only older adults showed significantly lower CST accuracy scores in the dual- compared to single-task conditions across both SNRs. Both older and younger adults showed significantly greater SD of lane position in the dual- compared to the single-task conditions, only under high driving load. Findings may inform educational practices, policies, and technological solutions to maintain and support safe driving among older adults.

